# PERCEPTIONS OF EMPOWERMENT AMONG HOME CARE AIDES

**DOI:** 10.1093/geroni/igz038.2588

**Published:** 2019-11-08

**Authors:** Sandra S Butler, Nancy Kusmaul

**Affiliations:** 1 University of Maine, Orono, Maine, United States; 2 University of Maryland, Baltimore County, Baltimore, Maryland, United States

## Abstract

Workforce issues in eldercare are of growing concern with the dramatic aging of our population and the growing need for personal care assistance. Due to difficult job conditions, recruitment and retention of direct care workers have presented challenges throughout the country both in facility-based and community-based care. The empowerment of workers has been theorized as one vehicle for improving job satisfaction among healthcare workers. Models of structural and psychological empowerment have been explored as factors to reduce job strain among healthcare workers in institutional settings, but have not been well examined in the home care setting. This paper will report on an exploratory study of perceptions of empowerment among home care aides (HCAs) in two states. In-person and telephone semi-structured interviews were conducted with 12 HCAs ranging in age from 20 to 64; eight were white and U.S. born and four were black and African born. All 12 study participants reported positive aspects of empowerment in their home care work (e.g., receiving information and good support, feeling competent, having an impact on their clients’ lives, and finding the work meaningful), and most felt autonomous in their work and believed they had the resources they needed to do the job. Nonetheless, negative aspects of empowerment were also frequently described (e.g., poor support, constrained information and resources, limited autonomy due to regulations, and few opportunities for advancement). This paper will explore and interpret these seemingly contradictory findings, with a discussion of implications for recruitment and retention of this valuable workforce

